# 6. *Staphylococcus aureus* in a Single Blood Culture Bottle: Should We be Concerned?

**DOI:** 10.1093/ofid/ofab466.006

**Published:** 2021-12-04

**Authors:** John Raymond U Go, Douglas Challener, Cristina G Corsini Campioli, Muhammad R Sohail, Raj Palraj, Larry M Baddour, Omar Abu Saleh

**Affiliations:** 1 Mayo Clinic Rochester, Rochester, MN; 2 Mayo Clinic, Rochester, Minnesota; 3 Baylor College of Medicine, Houston, Texas; 4 Mayo Clinic College of Medicine, Rochester, MN

## Abstract

**Background:**

*Staphylococcus aureus* bacteremia (SAB) is common and is characterized by high rates of morbidity and mortality. The clinical importance of a single positive blood culture bottle (SPBCB), however, is poorly defined despite it being a frequent laboratory finding. We therefore examined patients with SPBCB to determine its clinical significance and to understand the rationale of current practice.

**Methods:**

We performed a retrospective, multicenter study of patients with a SPBCB for *S. aureus* in initial cultures from January 2019 to December 2019 using data collected from both electronic health records and the clinical microbiology laboratory.

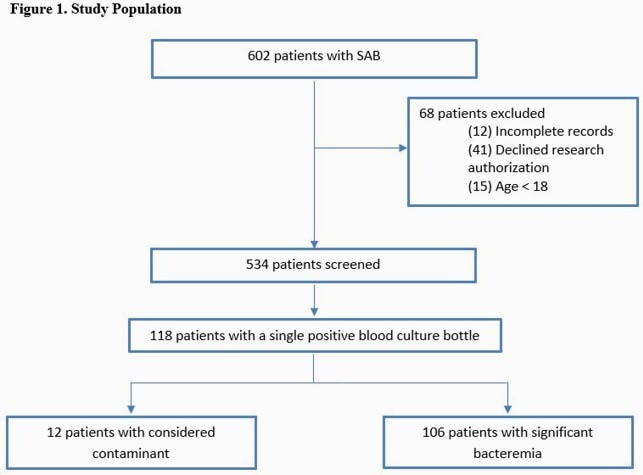

**Results:**

Overall, 534 patients with SAB were identified, and 118 (22.1%) had a SPBCB. Among SPBCB cases, 106 (89.3%) were classified as clinically significant while 12 were considered contaminated or of unclear clinical significance. Baseline characteristics were similar between the groups (Table 1). A majority (92.4%) received antibiotic therapy, but patients with clinically significant bacteremia were treated with a longer antibiotic course (25.9 vs 5.7 days, p< 0.001). Outcomes between those with SPBCB (contaminant vs clinically significant) were similar (Table 2). Of note, while there was no difference in use of echocardiography based on PREDICT criteria between the clinically significant SPBCB vs. the multiple positive blood culture bottles (MPBC) cohorts (Table 3), significant differences were seen in both frequency of echocardiography (65.1% vs. 84.6%, P< 0.001) and IE diagnosis (3.8% vs. 14.2%, P=0.002) for patients in the SPBCB vs. MPBC groups, respectively. In addition, those with MPBC had higher 90-day, 6-month and 1-year mortality rates.

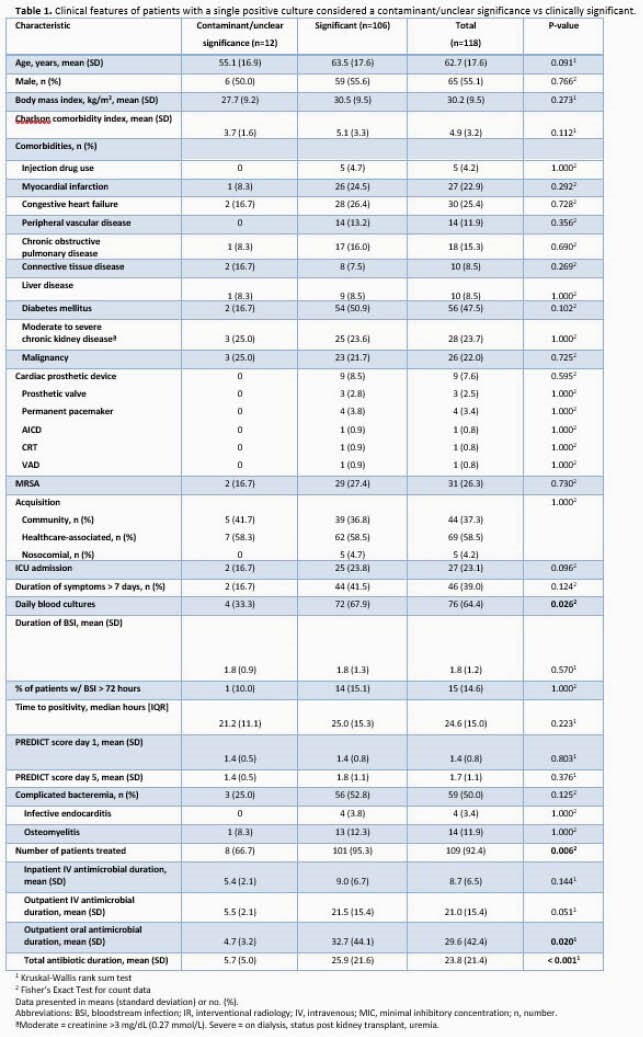

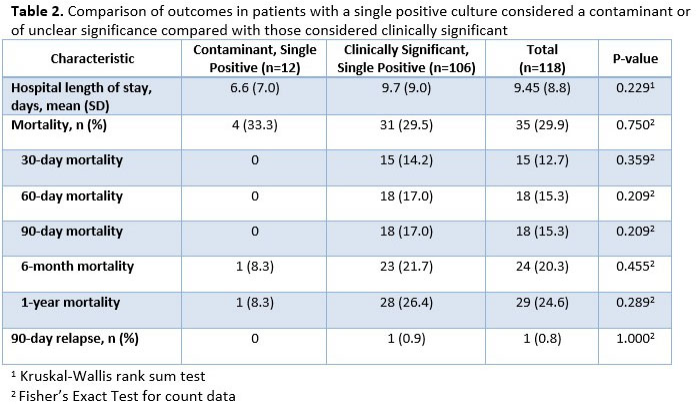

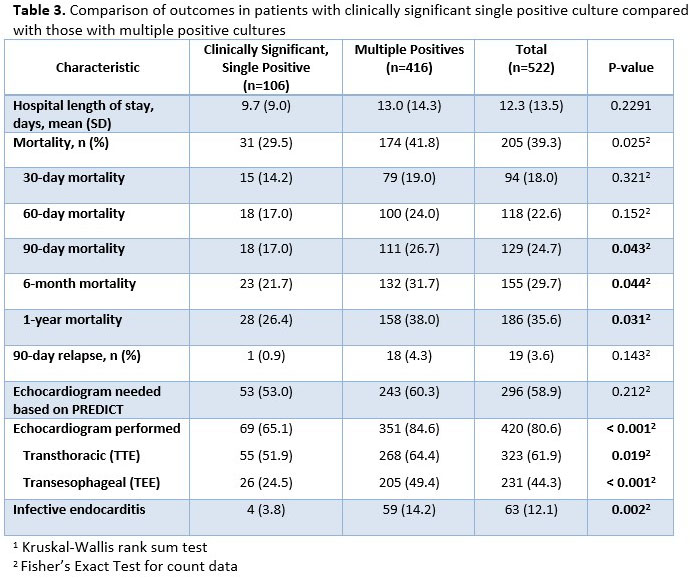

**Conclusion:**

SPBCB was documented in almost one-quarter of SAB cases and should trigger a thorough investigation as its associated mortality was high and complications, including IE, occurred. Although some SPBCB cases may represent contamination, antibiotic treatment of SPBCB was commonplace. Patients with clinically significant SPBCB were less likely to undergo echocardiography and had a reduced prevalence of an IE diagnosis as compared to those with MPBC. Patients with SPBCB may have a more favorable long-term prognosis as compared to that in patients with MPBC.

**Disclosures:**

**Muhammad R. Sohail, MD**, **Medtronic** (Consultant)**Philips** (Consultant) **Larry M. Baddour, MD**, Boston Scientific (Individual(s) Involved: Self): Consultant; Botanix Pharmaceuticals (Individual(s) Involved: Self): Consultant; Roivant Sciences (Individual(s) Involved: Self): Consultant

